# Ustekinumab Offers Long-Term Clinical Remission With Safety in Very Early-Onset Inflammatory Bowel Disease

**DOI:** 10.1093/ibd/izae078

**Published:** 2024-04-08

**Authors:** Itaru Iwama, Masashi Yoshida, Ayako Miyazawa, Tomoko Hara, Ryusuke Nambu

**Affiliations:** Division of Gastroenterology and Hepatology, Saitama Children’s Medical Center, Saitama, Japan; Division of Gastroenterology and Hepatology, Saitama Children’s Medical Center, Saitama, Japan; Division of Gastroenterology and Hepatology, Saitama Children’s Medical Center, Saitama, Japan; Division of Gastroenterology and Hepatology, Saitama Children’s Medical Center, Saitama, Japan; Division of Gastroenterology and Hepatology, Saitama Children’s Medical Center, Saitama, Japan

**Keywords:** ustekinumab, very early-onset inflammatory bowel disease, steroid-free clinical remission

Very early-onset inflammatory bowel disease (VEO-IBD) accounts for 1% to 15% of childhood inflammatory bowel disease (IBD) cases and is often refractory to medical therapy.^1,2^ Ustekinumab (UST), a human monoclonal antibody directed against P40, a subunit of interleukin-12 and interleukin-23, is approved for treatment of refractory ulcerative colitis (UC) and Crohn’s disease (CD). The efficacy and safety of UST in IBD have been reported in adults and older children^3-5^ but not yet in VEO-IBD patients.

Eight VEO-IBD patients (3 female) were treated with UST (Table 1). Diagnoses based on the revised Porto criteria^6^ were UC in 2, CD in 4, and unclassified IBD in 2. The median age at IBD diagnosis was 4.5 years, The median interval between diagnosis and UST initiation was 3.5 months. Six patients had a comorbidity. Mutations known to cause monogenic IBD were excluded by next-generation sequencing analysis. Seven patients had been treated with steroids and immunomodulators, 6 with 1 biologic agent, and 2 with 2 biologic agents. Six were receiving steroids at the time of UST initiation. The median initial UST dose was 8 mg/kg, while the median maintenance dose was 2.4 mg/kg. Disease severity at UST initiation was low or moderate in all patients. Steroid-free clinical remission (SFCR) rates at 26 and 52 weeks were 50% (n = 4 of 8), improving to 88% (n = 7 of 8) at 104 weeks. Clinical remission (CR) rates were 38% (n = 3 of 8), 63% (n = 5 of 8), and 75% (n = 6 of 8) at 8, 26, and 52 weeks, respectively. No serious side effects were observed.

**Table 1.** Characteristics and outcomes for 8 VEO-IBD patients undergoing UST therapy

	Sex	Diagnosis	Comorbidity	Age at diagnosis (y)	Previous treatment	Age UST begun (y)	Months from diagnosis to starting UST	Disease severity at UST initiation	UST dose for induction therapy (mg/kg)	UST dose for maintenance therapy (mg/kg)	Duration of UST use (mo)	Outcome		Side effects
												52 wk	Long term	
1	F	CD	9p syndrome	5	PSL, AZA, IFX	6	8	PCDAI 7.5	13	3.8	27	CR with low-dose steroid	SFCR for 2.3 y	None
2	M	CD	None	1	IFX	1	2	PCDAI 27.5	11.8	2	28	SFCR	SFCR for 2.3 y	None
3	F	UC	Hypoxic encephalopathy	5	PSL, AZA, ADA	6	3	PUCAI 10	7	2.4	31	SFCR	SFCR for 2.6 y	None
4	M	IBD-U	Snyder-Robinson syndrome	3	PSL, AZA, ADA	3	2	PUCAI 40	7.5	1.7	34	Mild activity without steroid	SFCR for 2.8 y	None
5	F	CD	Schaaf-Yang syndrome	3	PSL, AZA, IFX	4	6	PCDAI 10	6.7	2.5	56	CR with low-dose steroid	SFCR for 4.7 y	None
6	M	IBD-U	Dandy-Walker syndrome	5	PSL, AZA, ADA, IFX	5	3	PUCAI 35	7	2.4	34	Mild activity without steroid	SFCR for 2.8 y	None
7	M	CD	None	4	PSL, AZA, ADA, IFX	4	4	PCDAI 37.5	8.6	3	49	SFCR	SFCR for 4.1 y	None
8	M	UC	Congenital myopathy	5	PSL, AZA	7	30	PUCAI 40	10	3.2	21	SFCR	SFCR for 1.8 y	None

Abbreviations: ADA, adalimumab; AZA, azathioprine; CD, Crohn’s disease; CR, clinical remission; F, female; IBD-U, unclassified inflammatory bowel disease; IFX, infliximab; M, male; PCDAI, Pediatric Crohn’s Disease Activity Index; PSL, prednisolone; PUCAI, Pediatric Ulcerative Colitis Activity Index; SFCR, steroid-free clinical remission; UC, ulcerative colitis; UST, ustekinumab; VEO-IBD, very early-onset inflammatory bowel disease.

VEO-IBD has been reported to be more resistant to biologic agents than IBD with later onset. Bramuzzo et al^7^ reported a CR rate of 15.4% at 1 year after initiation in 42 VEO-IBD patients treated with infliximab, significantly lower than in older children. A report of 16 children given vedolizumab for VEO-IBD showed CR in 56% at 14 weeks after initiation, but long-term efficacy of vedolizumab is unknown.^8^ Efficacy of UST in pediatric IBD has been reported mainly in older children, with SFCR at 1 year after initiation of 46% and 45% in UC and CD, respectively^9^—a frequency similar to an SFCR in VEO-IBD in this study. Thus, UST in VEO-IBD showed efficacy comparable to that seen in older children, suggesting potential for long-lasting efficacy. Multicenter studies involving larger numbers of patients will be needed.

## Data Availability

The raw data supporting the conclusions of this article will be made available by the authors, with no undue reservations.
